# Evaluation of self‐collected nasal, urine, and saliva samples for molecular detection of SARS‐CoV‐2 using an EUA approved RT‐PCR assay and a laboratory developed LAMP SARS‐CoV‐2 test

**DOI:** 10.1002/iid3.1285

**Published:** 2024-06-18

**Authors:** Ana Purcell‐Wiltz, Fernando Tadeu Zamuner, Karem Caraballo, Lorena De Jesus, Yaima Miranda, Denise Ortiz, Amanda García Negrón, Andrea Cortés Ortiz, Adriana Baez, Josefina Romaguera, Ivonne Jiménez, Alberto Ortiz, Jorge Acevedo, Liliana Viera, David Sidransky, Rafael Guerrero‐Preston

**Affiliations:** ^1^ Biomarker Discovery and Validation Laboratory, LifeGene‐Biomarks Toa Baja Puerto Rico; ^2^ Internal Medicine Department San Juan Bautista School of Medicine Caguas Puerto Rico; ^3^ Otolaryngology Department, Head and Neck Cancer Research Division Johns Hopkins University, School of Medicine Baltimore Maryland USA; ^4^ Otolaryngology Department University of Puerto Rico School of Medicine San Juan Puerto Rico; ^5^ Obstetrics and Gynecology Department University of Puerto Rico School of Medicine San Juan Puerto Rico; ^6^ Internal Medicine Department University of Puerto Rico School of Medicine San Juan Puerto Rico; ^7^ Department of Surgery University of Puerto Rico School of Medicine San Juan Puerto Rico

**Keywords:** COVID‐19, invasive, LAMP, LDT, nasal swabs, RT‐PCR, saliva, SARS‐CoV‐2, urine

## Abstract

As the SARS‐CoV‐2 virus spread throughout the world, millions of positive cases of COVID‐19 were registered and, even though there are millions of people already vaccinated against SARS‐CoV‐2, a large part of the global population remains vulnerable to contracting the virus. Massive nasopharyngeal sample collection in Puerto Rico at the beginning of the pandemic was limited by the scarcity of trained personnel and testing sites. To increase SARS‐CoV‐2 molecular testing availability, we evaluated the diagnostic accuracy of self‐collected nasal, saliva, and urine samples using the TaqPath reverse transcription polymerase chain reaction (RT‐PCR) COVID‐19 kit to detect SARS‐CoV‐2. We also created a colorimetric loop‐mediated isothermal amplification (LAMP) laboratory developed test (LDT) to detect SARS‐CoV‐2, as another strategy to increase the availability of molecular testing in community‐based laboratories. Automated RNA extraction was performed in the KingFisher Flex instrument, followed by PCR quantification of SARS‐CoV‐2 on the 7500 Fast Dx RT‐PCR using the TaqPath RT‐PCR COVID‐19 molecular test. Data was interpreted by the COVID‐19 Interpretive Software from Applied Biosystems and statistically analyzed with Cohen's kappa coefficient (k). Cohen's kappa coefficient (k) for paired nasal and saliva samples showed moderate agreement (0.52). Saliva samples exhibited a higher viral load. We also observed 90% concordance between LifeGene‐Biomarks' SARS‐CoV‐2 Rapid Colorimetric LAMP LDT and the TaqPath RT‐PCR COVID‐19 test. Our results suggest that self‐collected saliva is superior to nasal and urine samples for COVID‐19 testing. The results also suggest that the colorimetric LAMP LDT is a rapid alternative to RT‐PCR tests for the detection of SARS‐CoV‐2. This test can be easily implemented in clinics, hospitals, the workplace, and at home; optimizing the surveillance and collection process, which helps mitigate global public health and socioeconomic upheaval caused by airborne pandemics.

## Graphical abstract

The objective of this study was to evaluate the diagnostic accuracy of the TaqPath reverse transcription polymerase chain reaction (RT‐PCR) COVID‐19 kit in paired nasopharyngeal, saliva, and urine samples, as well as a loop‐mediated isothermal amplification (LAMP) SARS‐CoV‐2 laboratory developed test (LDT) in nasopharyngeal samples. Our findings suggest that utilizing saliva samples for RT‐PCR TaqPath and LifeGene‐Biomarks SARS‐CoV‐2 rapid colorimetric LAMP LDT holds promise as an effective alternative method for diagnosing COVID‐19.

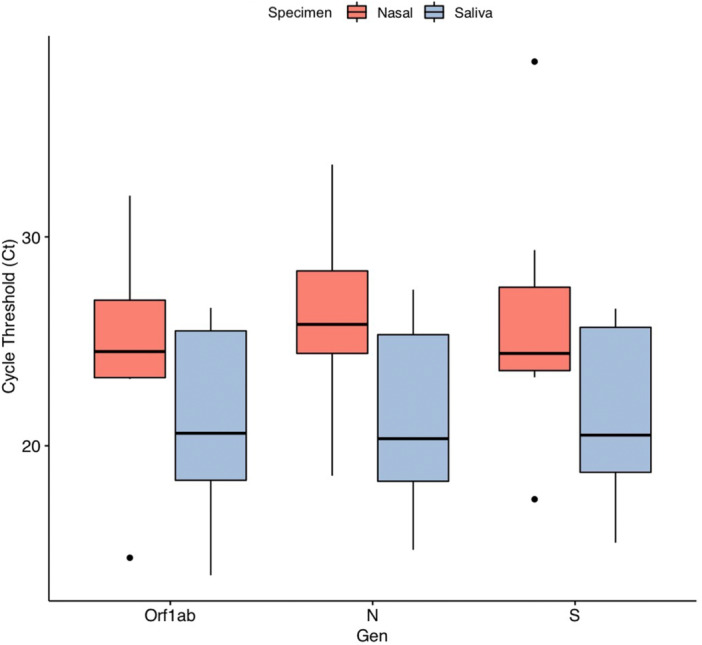

## 1 INTRODUCTION

In early 2020, the World Health Organization (WHO) declared a new viral pandemic for coronavirus disease 2019 (COVID‐19), an infection caused by severe acute respiratory syndrome SARS‐CoV‐2 that was identified in Wuhan, China.^1^ In just a few months, COVID‐19 spread throughout the world, causing more than 103 million positive cases in the United States and 1 million positive cases in Puerto Rico.^2^ Our understanding of the SARS‐CoV‐2 virus and COVID‐19 disease has significantly improved since the onset of the pandemic due to extensive research that has enabled more effective diagnostic strategies, treatments, and preventive measures.^3^ However, despite our growing understanding, there are still ongoing efforts to elucidate various aspects of SARS‐CoV‐2 behavior, disease progression, and the ever‐evolving landscape of diagnostic methodologies. Reverse transcription polymerase chain reaction (RT‐PCR) is the most widely used molecular diagnostic test for the detection of the virus in biological samples, and this includes assays such as TaqPath, which have provided a robust platform for viral detection across various laboratories.^4^ Selection of the appropriate test for sample collection is very important to obtain reliable results. Nasal and oropharyngeal swabs have proven to be the most reliable specimen types for both RT‐PCR and antigen tests.^5^ Although these swabs are relatively easy to collect and the test results are very sensitive, there are limitations related to their collection, scarcity on the island, and the safety of healthcare personnel.^6^ For this reason, the use of saliva has been suggested as alternatives to nasal swabs for the detection of SARS‐CoV‐2.^7^

SARS‐CoV‐2 has been shown to persist on a variety of surfaces for up to 9 days under experimental conditions^8^ and in various locations in hospital rooms.^9^ This effect is due to the continuous dissemination of millions of copies of the virus by subjects for up to 37 days,^9^, ^10^ from droplets that are released from the nose and mouth when coughing, sneezing or speech. Consequently, SARS‐CoV‐2 has been shown to spread from person to person through close contact and respiratory droplets, as well as contact with contaminated surfaces and objects.^11^ In addition, since the incubation period of SARS‐CoV‐2 is longer than that of influenza,^12^ SARS‐CoV‐2 infection has spread rapidly throughout the world, and it is already among the worst pandemics in history.^13^, ^14^ Although current data indicate variations in disease outcomes, with some regions experiencing lower severity and mortality rates,^15^ continued vigilance and research are crucial to comprehensively understand the factors influencing disease severity and to ensure appropriate public health responses.

COVID‐19 and influenza have a similar disease presentation because both are transmitted by physical contact and cause respiratory illness that presents in a wide range of symptoms such as fever and dry cough.^11^ For healthcare workers and hospitals, this situation could pose a challenge when diagnosing the disease and, therefore, there is considerable demand for rapid and affordable diagnostic tests.^16^ COVID‐19 is diagnosed primarily through quantitative PCR testing based on Ct values that correlate with the SARS‐CoV‐2 viral load, its replication and transmissibility.^17^ However, considering the main modes of transmission of SARS‐CoV‐2, different screening strategies are being adopted and there is not a universally accepted test.^17^, ^18^, ^19^, ^20^, ^21^ Furthermore, genomic surveillance algorithms have been created by precision health diagnostic and surveillance networks as a preventive measure for the spread of SARS‐CoV‐2 variants of concern.^17^ The algorithms mentioned are a tool for quick tracking of highly infectious variants of COVID‐19 and could work alongside a rapid, universally accepted test, to contain viral spread.

Saliva and urine diagnostics can play a fundamental role in the detection of COVID‐19 as they are noninvasive, inexpensive, safe, and require less handling than other tests such as nasopharyngeal and oropharyngeal swabs and blood tests.^22^, ^23^, ^24^ On another hand, the collection of respiratory tract samples is more limited because it requires technical skills to collect them and protective equipment for the healthcare workers.^24^ It is recognized that these tests are essential to help inform individual quarantine efforts and prevent spread, as well as to help track what cities are more susceptible and at higher risk.^4^

The COVID‐19 pandemic has resulted in a significant demand for reliable diagnostic tests that can quickly detect SARS‐CoV‐2, the virus responsible for the disease. Among the most widely used testing methods are RT‐PCR and loop‐mediated isothermal amplification (LAMP) tests. The TaqPath RT‐PCR COVID‐19 Kit (Thermo Fisher Scientific) is one of the many diagnostic kits that have been granted Emergency Use Authorization (EUA) by the FDA.^21^, ^25^, ^26^ The kit is designed to detect the RNA from SARS‐CoV‐2 in respiratory specimens from patients suspected of having COVID‐19. LAMP tests, on the other hand, are an alternative to RT‐PCR tests, and they have gained popularity due to their simplicity and faster turnaround time.^27^

While waiting for the citizen vaccination process to conclude to reach herd immunity and combat COVID‐19, it is of the utmost importance to diagnose it correctly to stop its spread, especially for asymptomatic cases. In this study, the feasibility of using self‐collected saliva, nasal, and urine samples to detect the viral load of SARS‐CoV‐2 was examined. We compared COVID‐19 polymerase chain reaction (PCR) test results with an EUA in saliva, urine, and nasal specimens of positive and negative subjects for SARS‐CoV‐2. We also tested LifeGene‐Biomarks SARS‐CoV‐2 RT‐LAMP Diagnostic Assay, a LAMP assay, for the qualitative detection of nucleic acid from SARS‐CoV‐2 in nasopharyngeal swabs from individuals suspected of COVID‐19 by a healthcare provider. The integration of saliva, nasal, and urine molecular testing, as well as public surveillance efforts, contribute to the project's goals by alleviating the current global COVID‐19 pandemic and guiding responses to future outbreaks worldwide.

Given the difficulty of nasopharyngeal sample collection for SARS‐CoV‐2 testing in large scale and the lack of SARS‐CoV‐2 molecular tests at the beginning of the pandemic, we implemented a Biomarker Development Trial to evaluate the use of self‐sampling for SARS‐CoV‐2 testing in nasal, urine, and saliva samples. We also evaluated the diagnostic accuracy of a rapid LAMP SARS‐CoV‐2 Laboratory Developed Test using the TaqPath RT‐PCR COVID‐19 kit as the reference method.

## 2 MATERIALS AND METHODS

We conducted a prospective study to identify which self‐collected method would provide sensitive COVID‐19 PCR results without requiring sample collection by a medical technologist. All study subjects were represented by a unique study ID and not by name. Although subjects are asked to complete questionnaires at the start of the study, they were instructed that they could choose not to answer any question(s) they did not wish to answer. The study coordinators responsible for completing questionnaires and gathering the required information were CITI certified and well‐versed in HIPAA requirements.

We obtained paired self‐collected nasal, saliva, and urine samples from 277 patients in Puerto Rico; all of which were collected especially for this study. The samples were collected in sterile devices and transported to the laboratory within 24 h of collection. The TaqPath RT‐PCR COVID‐19 kit (Thermo Fisher Scientific) was used to test SARS‐CoV‐2 nucleic acids in nasal, saliva and urine samples. Additionally, a total of 39 nasopharyngeal samples were used to develop a LAMP LDT. These 39 nasopharyngeal specimens are not part of the 277 paired samples mentioned prior. This study was approved by the Institutional Review Board (IRB), as it followed bioethics protocols in compliance with federal and applicable HIPAA privacy and security regulations (Prot. 2770120). Access to all collected data was limited to only the IRB approved study staff to safeguard confidentiality.

### 2.1 TaqPath RT‐PCR COVID‐19 kit (Thermo Fisher Scientific)

**Figure 1:**
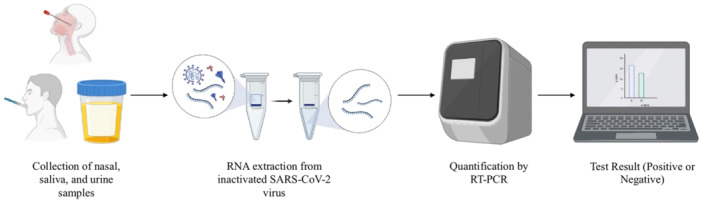
Process flowchart for nasal, urine, and saliva samples analysis using the TaqPath reverse transcription polymerase chain reaction (RT‐PCR) COVID‐19 kit.

The collection of nasal, urine, and saliva samples from patients over 21 years old was carried out in the Emergency Room of Puerto Rico Medical Center between June 23, 2020, and September 1, 2020. The samples were stored in −80 feezers at LifeGene‐Biomarks laboratory in Toa Baja, Puerto Rico. Automated RNA extraction was performed in the KingFisher Flex instrument (ThermoFisher), and SARS‐CoV‐2 PCR quantification in the 7500 Fast Dx RT‐PCR Instrument (Applied Biosystems), using the molecular test “TaqPath RT‐PCR COVID‐19 COMBO Kit” (ThermoFisher). The objective was to obtain data that can be used to compare the results of SARS‐CoV‐2 PCR in saliva with the results of SARS‐CoV‐2 PCR in nasal mucosa and urine samples (Figure 1).

The performance of a PCR test for molecular diagnosis of SARS‐CoV‐2 was compared using the “TaqPath RT‐PCR COVID‐19 COMBO kit” from ThermoFisher, approved by the FDA, in samples of nasal mucosa, urine, and saliva from SARS‐CoV‐2 positive and negative subjects in an incident case study. Therefore, this study was observational and no intervention or alteration to the course of the disease was performed. TaqPath is a multiplex real‐time reverse transcription PCR (RT‐PCR) test that uses primers and probes designed to detect the RNA of the SARS‐CoV‐2 virus. TaqPath amplifies regions of the N, S and ORF1ab genes of SARS‐CoV‐2.

In a real‐time PCR test, a positive reaction is detected by quantifying a fluorescent signal. TaqPath PCR results were analyzed using Applied Biosystems' COVID‐19 interpretive program. This program tabulates the Ct values for both the positive and negative controls for each run as well as for the collected samples, indicates the validity of the results, and provides a printed report containing the interpretation of the results. Ct values are defined as the number of cycles required for the fluorescent signal to cross the threshold. Ct values are also inversely proportional to the amount of nucleic acid in the sample.

### 2.2 Statistical analysis

Cohen's kappa coefficient (k) was used to compare the agreement between the TaqPath results in nasal, urine, and saliva samples. Cohen's kappa coefficient is a statistic that measures the inter‐rater reliability, or the degree of agreement, between two independent raters. It adjusts the effect of chance in the proportion of concordance observed for qualitative elements.^28^, ^29^, ^30^, ^31^ Significance was evaluated by paired test with Welch adjustment. R (version 4.0.4) and Microsoft Excel programs were used to perform all the biostatistical analyses.

### 2.3 LifeGene‐Biomarks SARS‐CoV‐2 LAMP laboratory developed test

Clinical Laboratory Improvement Amendments (CLIA) regulatory requirements were followed to develop an isothermal SARS‐CoV‐2 Laboratory Developed Test (LDT) in saliva using the SARS‐CoV‐2 Rapid Colorimetric LAMP Assay Kit (NEB, Cat. No. E2019S), a commercially available Research Use Only colorimetric LAMP Kit. An LDT is a single site test that belongs to the laboratory that develops it. It must undergo a research and development process that involves fundamental analytical validation tests to demonstrate that the assay satisfies clinical needs, standard operating procedures (SOPs), and the testing platforms that are accessible.^32^, ^33^ To be CLIA certified, LifeGene‐Biomarks SARS‐CoV‐2 LAMP Laboratory Developed Test adhered to laboratory quality management system (QMS) regulations. LAMP technology is a qualitative, sensitive, and affordable molecular method that detects amplified nucleic acids using isothermal amplification. This test uses two SARS‐CoV‐2 specific primer sets, designed to uniquely detect SARS‐CoV‐2 RNA. Laboratorio Clínico del Mar II in Vega Baja, Puerto Rico implemented LifeGene‐Biomarks’ SARS‐CoV‐2 Rapid Colorimetric LAMP LDT in nasopharyngeal samples that were already tested with RT‐PCR. The objective was to compare the positive or negative result of the RT‐PCR with the positive or negative result of LifeGene‐Biomarks SARS‐CoV‐2 Rapid Colorimetric LAMP LDT. All results were concealed throughout the course of the experiment. LifeGene‐Biomarks also developed SARS‐CoV‐2 Rapid Colorimetric LAMP LDT for saliva samples, but it was not used in a clinical laboratory setting.

**Figure 2:**
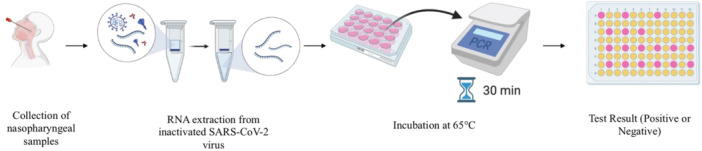
Process flowchart for nasopharyngeal samples analysis loop mediated amplification (LAMP) analysis.

First, in addition to the 277 paired samples previously described, 39 nasopharyngeal specimens were collected and transported in appropriate transport media. Samples were then transferred to a 96‐well plate for bead‐based automated RNA extraction using the KingFisher Flex (Fisher Scientific). RNA was extracted using the Viral MagMAX Viral/Pathogen Nucleic Acid Isolation Kit (Applied Biosystems) and was then transferred from the extraction elution plate to a 96‐well plate where the reaction was set up for incubation. The 96‐well plates were incubated at 65°C for 30 min. During this isothermal reaction, reverse transcription and loop‐mediated amplification occurred (Figure 2).

Extracted RNA was processed through the colorimetric procedure using three different primer sets: one targeting the SARS‐CoV‐2 nucleocapsid (N) gene, one targeting the SARS‐CoV‐2 envelope gene (E), and one targeting the human actin (rActin) gene. Each primer set is composed of six individual primers, targeting specific regions of viral or human RNA which are amplified during isothermal incubation using a strand‐displacing polymerase. The incorporation of dNTPs during amplification causes a pH change in the reaction which is visually detectable with pH‐sensitive dyes. The reaction color change initiated by amplification is visually measured after the 30 min incubation period.

## 3 RESULTS

### 3.1 Characteristics of the 277 patients undergoing COVID‐19 testing by TaqPath method

Table 1 gathers demographic information, lifestyle choices, health history, and includes COVID‐19 symptoms (if present) of these patients. Of the 277 patients, 52% were female, with a median age of 42 years (range 18‐82 years), 46% were single, 93% had a bachelor's degree, 90% were born in Puerto Rico, 41% were previously infected by influenza virus, 84% suffered from hypertension/elevated arterial pressure, and 65% consumed alcohol. When asked about their symptoms, 25% reported cough and 25% reported difficulty breathing. Additionally, 30% reported having contact with someone previously infected with COVID‐19.

**Table 1:** Characteristics of patients selected for this study.

	*n* = 277
	*n* (%)
**Age (years)**	
Median ± SD	42 ± 15
**Sex**	
Female	145 (52%)
Male	132 (48%)
**Marital status**	
Single	128 (46%)
Married	96 (34%)
Consensual union/lives with partner	17 (6%)
Separated	0
Divorced	3 (1%)
Refused to answer	13 (5%)
**Highest level of education**	
Middle School or less	15 (5%)
High School	61 (22%)
Vocational/technical school	4 (1%)
Currently attending university	7 (3%)
Some university studies	17 (6%)
Associate degree	52 (19%)
Bachelor's degree	93 (34%)
Graduate or professional degree	12 (4%)
Doctorate	14 (5%)
Refuses to answer	0
**Place of birth**	
Puerto Rico	248 (90%)
United States	10 (4%)
Dominican Republic	12 (4%)
Other country	4 (1%)
**Type of Infection**	**Been diagnosed with**
	***n*** (**%)**
Hepatitis C	4 (1%)
Hepatitis B	1 (0.4%)
Influenza	41 (15%)
Mycoplasma	23 (8%)
Human papillomavirus	5 (2%)
Human immunodeficiency virus	1 (0.4%)
**Chronic Illnesses**	**Been diagnosed with**
	***n*** (**%)**
Cardiac disease	18 (6%)
Hypertension/elevated arterial pressure	84 (30%)
Type 1 diabetes	1 (0.4%)
Type 2 diabetes	42 (15%)
Lupus	4 (1.4%)
Arthritis	22 (8%)
Crohn's disease	2 (1%)
Bronchial asthma	40 (14%)
Cancer	7 (3%)
Other	73 (26%)
**Lifestyle behavior**	***n*** (**%)**
Cigarette smoking	102 (37%)
Alcohol	179 (65%)
E‐Cigarette	14 (5%)
Vaping	17 (6%)
Medicinal cannabis	27 (10%)
Second ‐ hand smoking	46 (17%)
Other	12 (4%)
**COVID‐19 symptoms**	***n*** (**%)**
Cough	25 (9%)
Difficulty breathing	25 (9%)
Vomiting	11 (4%)
Diarrhea	16 (6%)
Fever >100°F	12 (4%)
Loss of taste	7(3%)
**COVID‐19 exposure**	***n* (%)**
Had contact with someone COVID positive	83 (30%)
Lives with someone with COVID symptoms	10 (4%)
Traveled recently	13 (5%)

### 3.2 Cohen's kappa coefficient (k) for nasal and saliva samples tested by TaqPath method

The saliva test (7.7%) detected a greater number of positives than the nasal test (4.4%). When calculating Cohen's kappa, of the 274 paired samples collected, 259 (94.5%) showed agreement. Some samples were discordant: 12 (4.5%) were saliva positive and nasal negative and 3 (1.1%) were saliva negative and nasal positive. Cohen's kappa coefficient (k) was 0.52, indicating moderate agreement between paired nasal and saliva samples.^28^, ^29^, ^30^, ^31^ Supplementary Table 1 shows the data of this calculated kappa statistic.

Once Cohen's kappa coefficient was calculated, the CI was calculated. Kappa = 0.52 with a 95% confidence interval was calculated and computed to a confidence interval of 0.28 to 0.76.

### 3.3 Cohen's kappa coefficient (k) for nasal and urine samples tested by TaqPath method

The nasal test (2.33%) detected a greater number of positives than the urine test (0.78%). When calculating Cohen's kappa, of the 257 paired samples collected, 249 (96.8%) showed agreement. Some samples were discordant: 6 (2.3%) were nasal positive and urine negative and 2 (0.78%) were nasal negative and urine positive. Cohen's kappa coefficient (k) was −0.012, indicating no agreement between paired nasal and urine samples.^28^, ^29^, ^30^, ^31^ Supplementary Table 2 shows the data of this calculated kappa statistic.

Once Cohen's kappa coefficient was calculated, the confidence interval (CI) was calculated. Kappa = −0.012 with a 95% confidence interval was calculated and computed to a confidence interval of −0.70 to 0.68.

### 3.4 Cohen's kappa coefficient (k) for saliva and urine samples tested by TaqPath method

The saliva test (5.5%) detected a greater number of positives than the urine test (0.74%). When calculating Cohen's kappa, of the 271 paired samples collected, 254 (93.7%) showed agreement. Several samples were discordant: 15 (5.5%) were positive for saliva and negative for urine and 2 (0.74%) were saliva negative and urine positive. Cohen's kappa coefficient (k) was −0.014, indicating no agreement between paired saliva and urine samples.^28^, ^29^, ^30^, ^31^ Supplementary Table 3 shows the data of the calculated kappa statistic.

Once Cohen's kappa coefficient was calculated, the CI was calculated. Kappa = −0.014 with a 95% confidence interval was calculated and computed to a confidence interval of −0.48 to 0.46.

### 3.5 Ct values (“cycle threshold”) for nasal and saliva samples tested by TaqPath method

Since nasal and saliva samples were the only ones that displayed concordance, their Ct values for ORF1ab, N, and S genes were compared. Out of 274 paired nasal and saliva samples collected, 9 were positive for COVID‐19 while 250 were negative. Table 2 shows the summary statistics and interquartile range (IQR) of ORF1ab, N, and S Ct values for the 9 paired positive samples.

**Table 2:** Summary of distribution and interquartile range of Ct values for ORF1ab, N and S.

Gen	Min	Q1	Median	Mean	Q3	Max	IQR
***Positive patients with nasal sample (n = 9)***							
**ORF1ab**	14.64	23.26	24.51	24.67	26.97	31.97	3.71
**N**	18.57	24.42	25.81	26.17	28.37	33.46	3.95
**S**	17.44	23.6	24.42	25.95	27.59	38.39	3.99
***Positive patients with saliva sample (n = 9)***							
**ORF1ab**	13.8	18.35	20.6	21.42	25.5	26.6	7.15
**N**	15.02	18.3	20.34	21.21	25.32	27.47	7.02
**S**	15.36	18.73	20.51	21.9	25.67	26.56	6.94

### 3.6 Paired *t* test for nasal and saliva samples tested by TaqPath method

When performing a paired *t* test,^34^ it was determined that there is no significant difference between the means of the Ct values for the ORF1ab gene (*p* = .19) and the S gene (*p* = .12). However, the mean of the Ct values for the N gene (*p* = .05) demonstrated statistical significance.

**Figure 3:**
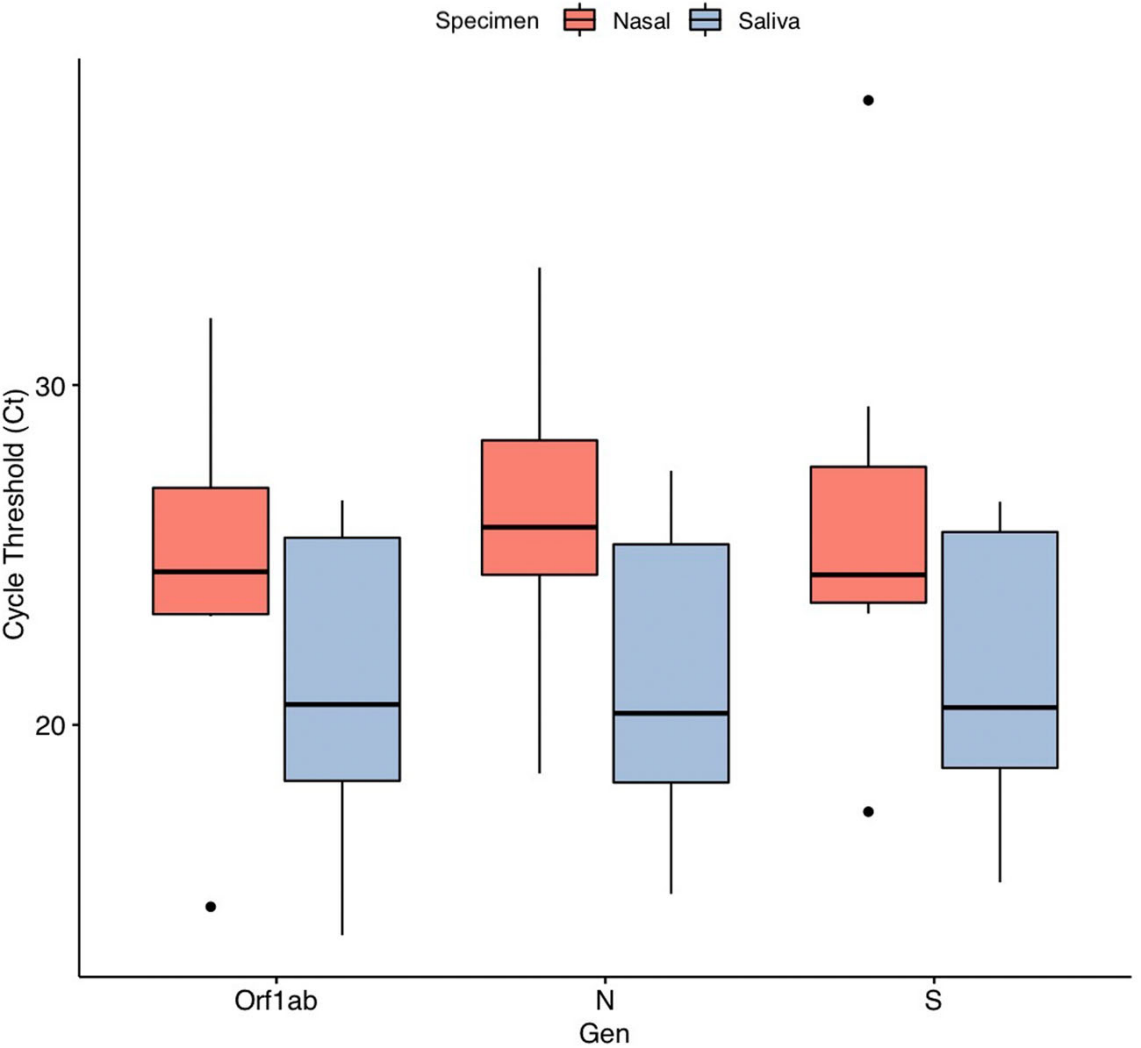
Box plots comparing Ct values of *ORF1ab*, *N*, and *S* in paired nasal and saliva samples positive for SARS‐CoV‐2.

Figure 3 shows a boxplot of Ct values for paired saliva and nasal swab samples positive to SARS‐CoV‐2. For the samples collected with saliva, the median Ct values for *ORF1ab* (21.42/24.67), *N* is (21.21/26.17), and *S* (21.90/25.95) were lower in saliva when compared to nasal swabs for all three SARS‐CoV‐2 genes, suggesting higher viral loads in saliva.

### 3.7 LifeGene‐Biomarks SARS‐CoV‐2 LAMP LDT

LifeGene‐Biomark's isothermal SARS‐CoV‐2 LDT optimized a commercially available colorimetric LAMP Kit (New England BioLabs). A total of 39 nasopharyngeal samples were collected and tested with RT‐PCR and LifeGene‐Biomark's isothermal SARS‐CoV‐2 LDT. These 39 nasopharyngeal specimens represent an additional set of samples and are not part of the 277 paired samples used for RT‐PCR TaqPath. Positive or negative results were concealed throughout the course of the experiment. Upon interpretation of the results from both testing methods, a concordance of 90% was observed.

## 4 DISCUSSION

Our results suggest that self‐collected saliva is superior to nasal and urine samples for COVID‐19 testing. The results also suggest that the colorimetric LAMP LDT is a rapid alternative to RT‐PCR tests for the detection of SARS‐CoV‐2. A colorimetric LAMP test can be easily implemented in clinics, hospitals, the workplace, and at home, optimizing the surveillance and collection process, to mitigate global public health and socioeconomic upheaval caused by airborne pandemics.

Saliva, nasal swabs, and urine have long been used as a diagnostic method for a variety of systemic diseases.^23^, ^35^ However, because saliva is noninvasive and easy to obtain, it has been extensively used by researchers in the past to search for biomarkers and diagnose a variety of oral diseases. This study evaluated the effectiveness of self‐collected nasal, urine, and saliva samples for molecular detection of SARS‐CoV‐2. Our objective was to evaluate the diagnostic accuracy of the TaqPath RT‐PCR COVID‐19 kit in paired nasopharyngeal, saliva, and urine samples and a LAMP SARS‐CoV‐2 Laboratory Developed Test in nasopharyngeal samples. Our findings suggest that utilizing saliva samples for RT‐PCR TaqPath and LifeGene‐Biomarks SARS‐CoV‐2 Rapid Colorimetric LAMP test holds promise as an effective alternative method for diagnosing COVID‐19.

Nasal swabs are widely accepted as samples for the detection of SARS‐CoV‐2 in the current pandemic. However, collection procedures cause discomfort, such as sneezing and coughing in most patients, generating droplets or aerosol particles that are dangerous to healthcare workers collecting these samples.^24^, ^36^ Discomfort in sample collection could also potentially contribute to patient failure to seek testing. Therefore, saliva and urine have been proposed as an alternative sample type for the diagnosis of COVID‐19. As both could be collected by patients themselves, it could reduce the risk of transmission to healthcare workers. Furthermore, the use of saliva and urine could reduce the demand for swabs. The evaluation of a diagnostic method is of utmost importance to guarantee its reproducibility in different epidemiological scenarios. This is particularly important in regions with lower income and access to resources, such as Puerto Rico, where this study was conducted.

In this study, a total of 277 paired nasal, saliva and urine samples were collected. Our aim was to evaluate the sensitivity of each collection method for COVID‐19 molecular testing, most specifically saliva. Our study is consistent with multiple published papers that support the use of saliva as an alternative sample for the diagnosis of COVID‐19,^7^, ^37^ and with one where saliva was shown to be more sensitive than the nasopharyngeal swab.^38^ Several reasons can potentially explain this affinity in the studies, such as the enrichment of oropharyngeal secretions, where the viral load is potentially higher, and/or the fact that saliva provides a larger volume (approximately 2 mL) of sample collection for analysis, and hence more RNA per sample.^39^

Cohen's kappa coefficient is a metric used to assess reliability between qualitative values. It is generally considered a more precise indicator than a simple percentage measurement of agreement, as it directly tests the probability of agreement occurring by chance.^28^, ^29^, ^30^ When comparing the concordance between nasal and saliva collection methods, Cohen's kappa was found to be 0.52, showing a moderate concordance. The 95% confidence interval ranges from a minimum of 0.28 (28% agreement) to a maximum of 0.76 (76% agreement). This result suggests that both nasal and saliva testing can detect SARS‐CoV‐2 infected individuals with high viral loads and have potential to determine highly contagious individuals, confirming the first hypothesis of the study. It should be noted that the standard error of the kappa coefficient (SEκ) is partially dependent on the sample size; the larger the number of observations measured, the smaller the expected standard of error.

When comparing the concordance between nasal and urine collection methods, Cohen's kappa resulted −0.012, indicating no agreement. The 95% confidence interval was calculated and was computed to an interval of a minimum −0.70 (−70% agreement) to a maximum of 0.68 (68% agreement). Saliva and urine collection methods also indicated no agreement, as their kappa coefficient resulted −0.014 with a 95% confidence of a minimum of −0.48 (−48% agreement) to a maximum of 0.46 (46% agreement). Therefore, our study suggests that urine is not a reliable collection method for detection of COVID‐19.

The number of cycles required to amplify viral RNA to a detectable level in an RT‐PCR assay is called the Ct. Thus, a Ct value was used to calculate the amount of viral RNA present in a sample. PCR tests work by amplifying the genetic material of the virus in cycles. The fewer cycles required, the greater the amount of virus, or viral load, per sample. Meanwhile, the higher the viral load, the greater the probability that the patient is contagious. Interpretation of the results of the TaqPath RT‐PCR COVID‐19 test was performed by the Applied Biosystems™ COVID‐19 Interpretive Software, a requirement set in the EUA approved for this assay. Supplementary Table 4 describes the result interpretation for patient samples and lists recommended actions per result. Supplementary Table 5 lists the Ct cutoff values for the assay targets. Since nasal and saliva samples were the only ones that displayed agreement, their Ct values for ORF1ab, N, and S genes were compared. A paired *t‐*test was performed for the 9 positive samples collected by saliva and nasal swab, to which the p‐value was found to be statistically significant only for difference in the N gene (*p* = .05). However, it was found that there were no significant differences between saliva and nasal Ct values for ORF1ab and S genes, suggesting similar viral loads in both samples.

Due to the significant difference for the N gene, when plotting the distribution of the Ct values, they turned out to be significantly lower in saliva than in nasal. These results suggest that saliva has even higher sensitivity for COVID‐19, which could allow for an easy collection process that is noninvasive. The origin of SARS‐CoV‐2 in saliva could come from multiple sources. Among them, the remnants of the nasopharyngeal epithelium that flow into the oral cavity and/or the salivary glands infected with SARS‐CoV‐2 that secrete the virus into the saliva.^38^

LDTs are diagnostic tests developed and performed by individual laboratories. LDTs are often used when commercial tests are not available or are insufficient for a particular application. LDTs are regulated by the CLIA program and must meet certain performance standards. Developing an LDT using the New England Biolabs Colorimetric LAMP kit to detect SARS‐CoV‐2 required obtaining the necessary equipment and reagents, validating the test's performance, and complying with regulatory requirements. Validation typically involves testing the LDT using known positive and negative samples, comparing the results to a reference method (such as RT‐PCR), and evaluating the test's sensitivity and specificity. The laboratory should also establish quality control measures to monitor the test's performance over time. LDTs have played a crucial role in expanding COVID‐19 testing capacity in the US, and continued development of LDTs will be important for controlling the spread of the disease, as it now enters the endemic phase. To test the optimized LDT, 39 nasopharyngeal samples were clinically analyzed with both the LDT and RT‐PCR. Out of the 39 samples, 35 obtained the same result in both the LDT and RT‐PCR, yielding 90% concordance. Upon calculation of the kappa coefficient, substantial concordance was determined as k = 0.79. Based on our observations, the LAMP‐LDT method appears to be a reliable alternative for SARS‐CoV‐2 detection, with results consistent with those obtained from RT‐PCR testing.

The diagnostic accuracy of TaqPath RT‐PCR COVID‐19 kit was evaluated in nine studies that included a total of 2357 paired samples. The sensitivity of TaqPath RT‐PCR COVID‐19 kit ranged from 84.5% to 100%, and the specificity ranged from 95.5% to 100% for the detection of SARS‐CoV‐2 in paired nasopharyngeal, saliva, and urine samples.^21^, ^25^, ^27^, ^40^, ^41^, ^42^, ^43^, ^44^, ^45^ The diagnostic accuracy of TaqPath RT‐PCR COVID‐19 kit was found to be higher in nasopharyngeal samples compared to saliva and urine samples. The accuracy of TaqPath RT‐PCR COVID‐19 kit was found to be affected by the viral load, sampling technique, and the time of sample collection after symptom onset. The diagnostic accuracy of LAMP SARS‐CoV‐2 Laboratory Developed Test was evaluated in four studies that included a total of 870 paired samples. The sensitivity of LAMP SARS‐CoV‐2 Laboratory Developed Test ranged from 76.9% to 100%, and the specificity ranged from 92.3% to 100% for the detection of SARS‐CoV‐2 in paired nasopharyngeal, saliva, and urine samples.^21^, ^25^, ^45^, ^46^ LAMP SARS‐CoV‐2 Laboratory Developed Test was found to be less sensitive than TaqPath RT‐PCR COVID‐19 kit, but its specificity was comparable to the TaqPath RT‐PCR COVID‐19 kit.

This study has some limitations. First, this is a single center (Emergency Room of Puerto Rico Medical Center) study. Second, the sample size was small, since of the 277 patients, only 9 had paired positive saliva and nasal samples for comparison. Nonetheless, the findings of this study are important because all nine participants were able to provide verifiable paired saliva and nasal samples. In conclusion, the use of saliva and LifeGene‐Biomarks SARS‐CoV‐2 Rapid Colorimetric LAMP test offer a promising alternative for the diagnosis of COVID‐19, as there is no universally accepted test for the detection of the virus in biological samples. Both are less invasive, do not require medical personnel for recollection, and can be beneficial in crowded settings such as the workplace. However, further evaluation is needed to explore their specificity and sensitivity in larger data sets.

## AUTHOR CONTRIBUTIONS

**Ana Purcell‐Wiltz**: Conceptualization; data curation; formal analysis; investigation; methodology; project administration; software; supervision; validation; visualization; writing—original draft; writing—review & editing. **Fernando Tadeu Zamuner**: Conceptualization; data curation; investigation; methodology; software; supervision; writing—review & editing. **Karem Caraballo**: Data curation; formal analysis; methodology. **Lorena De Jesus**: Data curation; formal analysis; methodology. **Yaima Miranda**: Data curation; formal analysis; methodology. **Denise Ortiz**: Data curation; investigation; methodology. **Amanda García Negrón**: Data curation; formal analysis; methodology. **Andrea Cortés Ortiz**: Investigation; writing—review & editing. **Adriana Baez**: Conceptualization; data curation; funding acquisition; methodology; project administration; supervision; writing—review & editing. **Josefina Romaguera**: Conceptualization; data curation; investigation; methodology; project administration; supervision; validation. **Ivonne Jiménez**: Conceptualization; data curation; methodology; project administration; supervision; writing—review & editing. **Alberto Ortiz**: Data curation; methodology; project administration. **Jorge Acevedo**: Data curation; methodology; supervision; writing—review & editing. **Liliana Viera**: Conceptualization; data curation; investigation; methodology; project administration; writing—review & editing. **David Sidransky**: Funding acquisition. **Rafael Guerrero‐Preston**: Conceptualization; data curation; formal analysis; funding acquisition; investigation; methodology; project administration; resources; software; supervision; validation; visualization; writing—original draft; writing—review & editing.

## Supporting information

Supporting information.

Supporting information.

Supporting information.

Supporting information.

Supporting information.

## Data Availability

RT‐PCR and LifeGene‐Biomarks SARS‐CoV‐2 Rapid Colorimetric LAMP data that support the findings of this study are available from the corresponding author upon reasonable request.
