# Supplementary material for: Evaluation of self‐collected nasal, urine, and saliva samples for molecular detection of SARS‐CoV‐2 using an EUA approved RT‐PCR assay and a laboratory developed LAMP SARS‐CoV‐2 test
Source: Immun Inflamm Dis. 2024 Jun 18;12(6):e1285. doi: 10.1002/iid3.1285 (PMC11184932; doi:10.1002/iid3.1285)
Supplement: Supplementary file 1 — Supporting information. [file IID3-12-e1285-s005.docx]

**Detection of Nucleic Acid from SARS‐CoV‐2**

TaqPath^TM^ COVID-19 Combo Kit (Applied Biosystems, Cat# A47814) was used to detect nucleic acid from SARS‐CoV‐2 by qualitative Multiplex real-time RT-PCR, according to the manufacturer’s instructions (revision F.0). Automated RNA extraction was performed using the KingFisher^TM^ Flex Magnetic Particle Processor with 96 Deep-Well Head and the MagMAX^TM^ Viral/Pathogen Nucleic Acid Isolation Kit (Cat# A42352) or MagMAX^TM^ Viral/Pathogen II Nucleic Acid Isolation Kit (Cat# A48383) with a sample input volume of 200 μL. Briefly, 4 KingFisher^TM^ Deepwell 96 Plates (Cat# A48305) labeled: “Wash 1” (Wash Buffer), “Wash 2” (80% Ethanol), “Elution Solution” and “Sample plate” were prepared. To prepare the “Sample plate", to each well was added: 5 μL of Proteinase K, 200 μL of sample, and 200 μL of Nuclease-free Water to the Negative Control well. Binding Bead Mix was previously prepared, 5 times gently mixed, added 275 μL to each sample and the Negative Control well. Then, added 5 μL of MS2 Phage Control to each sample well and the Negative Control well. The MVP_2Wash_200_Flex program was used on the KingFisher^TM^ Flex Magnetic Particle Processor with 96 Deep-Well Head (Cat# 5400630). After starting the run, the prepared plates were loaded into position when prompted by the instrument. After the run is complete, the “Elution Plate” was immediately removed from the instrument, then covered with MicroAmp^TM^ Clear Adhesive Film (Cat# 4306311). The samples were eluted in 50 μL of Elution Solution, placed on ice for immediate use in real-time RT‐PCR. The purified nucleic acid is reverse transcribed into cDNA and amplified using the TaqPath^TM^ RT-PCR COVID-19 Kit. To prepare the reaction mix, the following components sufficient for the number of RNA samples to be tested were combined, plus one Positive Control and one Negative Control: 6.25 μL of TaqPath^TM^ 1‐Step Multiplex Master Mix (No ROX^TM^) (4X), 1.25 μL of COVID-19 Real-Time PCR Assay Multiplex, 7.50 μL of Nuclease-free Water for a total reaction mix volume of 15.0 μL. Then added either 10 μL of purified sample RNA (from RNA extraction), 10 μL of Purified Negative Control, or 2 μL of Positive Control (25 copies/μL of TaqPath™ COVID‑19 Control) up to 25 μL of total volume to each well of the reaction plate. RT-PCR was performed using the Applied Biosystems^TM^ 7500 Fast Dx Real‐Time PCR Instrument, using SDS Software v1.4.1, with the following settings: Assay: Standard Curve (Absolute Quantitation), Run mode: Standard 7500, Passive reference: None, and Sample volume: 25 μL. The data was analyzed, then interpreted by the Applied Biosystems^TM^ COVID‐19 Interpretive Software (version 1.3).

**Interpretation of the results**

Interpretation of the results is performed by the Applied Biosystems™ COVID-19

Interpretive Software. For information about the Ct values that are used by the software to interpret results, see **Supplementary Table 5**.

Quality control and validity of results

A minimum of one Negative Control and one Positive Control must be present for each run. Additional Negative Control wells must be run for each extraction that is represented on a real-time RT-PCR plate. All control wells must pass for the real-time RT-PCR plate to be considered valid.

Validation of results is performed automatically by the Applied Biosystems™ COVID-19 Interpretive Software based on performance of the Positive and Negative Controls (**Supplementary Table 4**).

**Supplementary Table 4.** Result interpretation for patient samples

| ORF1ab | N gene | S gene | MS2 | | Status | Result | Action |
| --- | --- | --- | --- | --- | --- | --- | --- |
| NEG | NEG | NEG | | NEG | INVALID | NA | Repeat test.^[1]^ If the repeat result remains invalid, consider collecting a new specimen. |
| NEG | NEG | NEG | | POS | VALID | SARS-CoV-2  Not Detected | Report results to healthcare provider. Consider testing for other viruses. |
| Only one SARS-CoV-2 target = POS | | | | POS or NEG | VALID | SARS-CoV-2  Inconclusive^[1,2]^ | Repeat test.^[1]^ If the repeat result remains inconclusive, additional confirmation testing should be conducted if clinically indicated. |
| Two or more SARS-CoV-2 targets  = POS | | | | POS or NEG | VALID | Positive SARS- CoV-2 | Report results to healthcare provider and appropriate public health authorities. |

^[1]^ Retesting must be performed by re-extracting the original sample and repeating the RT-PCR.

^[2]^ Samples with a result of SARS-CoV-2 Inconclusive shall be retested one time.

**Supplementary Table 5.**  Ct cutoff values for assay targets

| Sample | Target | Ct cutoff |
| --- | --- | --- |
| Positive Control | MS2 | Valid Ct values are >37 |
|  | Viral targets | Valid Ct values are ≤S37 |
| Negative Control | MS2 | Valid Ct values are ≤S32 |
|  | Viral targets | Valid Ct values are >37 |
| Clinical samples | MS2 | Valid Ct values are ≤S32 |
|  | Viral targets | Positive Ct values are ≤S37 |
