# Supplementary material for: Evaluation of self‐collected nasal, urine, and saliva samples for molecular detection of SARS‐CoV‐2 using an EUA approved RT‐PCR assay and a laboratory developed LAMP SARS‐CoV‐2 test
Source: Immun Inflamm Dis. 2024 Jun 18;12(6):e1285. doi: 10.1002/iid3.1285 (PMC11184932; doi:10.1002/iid3.1285)
Supplement: Supplementary file 2 — Supporting information. [file IID3-12-e1285-s001.docx]

**Components of LifeGene Biomarkers SARS-CoV-2 LAMP LDT**

LifeGeneBiomarks SARS-CoV-2 Rapid Colorimetric LAMP Assay is a rapid colorimetric assay for in vitro detection of SARS-CoV-2 RNA that relies on loop-mediated isothermal amplification (LAMP). The WarmStart Colorimetric LAMP Master Mix with UDG combines WarmStart Bst 2.0 DNA Polymerase and WarmStart RTx Reverse Transcriptase to enable nucleic acid amplification at a single reaction temperature. The 2X colorimetric master mix also contains a weakly buffered solution and a pH-sensitive dye that changes color upon acidification. Successful amplification in the presence of target nucleic acids results in the production of protons that cause a decrease in pH, resulting in a clear visual color change from pink to yellow, or yellow/orange, that is easily detectable by eye.

The WarmStart activated enzymes allow dual control of enzyme activity by reversible, aptamer-based inhibition. This temperature-dependent activation helps prevent undesired non-specific priming and extension prior to isothermal incubation at 65°C, providing added security for setting up reactions at room temperature. The colorimetric mix is enabled with carryover prevention (dUTP/UDG). It is formulated with a mixture of dTTP and dUTP. This ensures both efficient isothermal amplification as well as the incorporation of dU into the reaction products. LAMP products containing dU serve as a substrate for Antarctic Thermolabile Uracil DNA Glycosylase (UDG) present in the master mix, allowing carryover contamination prevention. Antarctic Thermolabile UDG will be completely inactivated upon isothermal incubation at 65°C. Because LAMP can generate large quantities of DNA in very short periods of time, best practices to reduce contamination involve not opening LAMP reactions post amplification.

The SARS-CoV-2 LAMP Primer Mix provided within the kit contains a mixture of primers that target both the nucleocapsid (N) gene and envelope (E) gene of SARS-CoV-2. The primer sets perform well individually, but mixing them improves detection (<https://pubmed.ncbi.nlm.nih.gov/32635743>). Upon successful amplification, the reaction mix will change color from pink to yellow, or yellow/orange color. An internal control (IC) primer set that amplifies rActin is included to ensure the absence of inhibition from human nucleic acid templates. A positive control (PC) template containing the N-gene is also included. Guanidine hydrochloride has been shown to improve colorimetric LAMP at a concentration of 40 mM and is provided to supplement in the reaction for any samples that do not already contain guanidine. Guanidine concentrations up to 60 mM (final concentration at 1X) are tolerated. A sample can only be judged for the presence or absence of SAR-CoV-2 RNA if: all reactions are pink prior to incubation and post incubation, the NTC reaction is pink, the PC reaction is yellow and the IC reaction is yellow.
